# PD-L1^+^ lung cancer stem cells modify the metastatic lymph-node immunomicroenvironment in nsclc patients

**DOI:** 10.1007/s00262-020-02648-y

**Published:** 2020-08-17

**Authors:** A. Raniszewska, H. Vroman, D. Dumoulin, R. Cornelissen, J. G. J. V. Aerts, J. Domagała-Kulawik

**Affiliations:** 1 Department of Pathology, Medical University of Warsaw, Pawinskiego 7 Street, 02-106 Warsaw, Poland; 2 Department of Pulmonary Medicine, Erasmus MC Cancer Institute, s-Gravendijkwal 230, 3015 CE Rotterdam, The Netherlands; 3 Department of Internal Medicine, Pulmonary Diseases and Allergy, Medical University of Warsaw, Banacha 1a Street, 02-097 Warsaw, Poland

**Keywords:** Lung cancer, Cancer stem cells, EBUS/TBNA, Lymph nodes, T cells

## Abstract

**Introduction:**

Cancer stem cells (CSCs) are implicated in tumor initiation and development of metastasis. However, whether CSCs also affect the immune system is not fully understood. We investigated correlations between the PD-L1^+^ CSCs, changes in T-cell phenotype in metastatic and non-metastatic lymph nodes (LNs) and response to treatment.

**Methods:**

LNs’ aspirates were obtained during the EBUS/TBNA procedure of 20 NSCLC patients at different stages of the disease. CSCs and T-cell characteristics were determined by flow cytometry.

**Results:**

PD-L1^+^ CSCs positively correlated with the percentage of Tregs, PD-1^+^ CD4 T cells and Tim3^+^ CD4^+^ T cells, whereas PD-L1^+^ CSCs were negatively correlated with CD4^+^ T cells and CD28^+^ CD4^+^ T cells. The percentage of PD-L1^+^ CSCs was higher in patients with progressive disease (PD) as compared to patients with stable disease (SD) or partial response (PR). Among T cells, only PD-1^+^ CD4^+^ T cells and Tim3^+^ CD4^+^ T-cell frequencies were higher in patients with PD as compared to patients with SD or PR.

**Conclusion:**

The frequency of PD-L1^+^ CSCs associates with an altered T-cell frequency and phenotype indicating that CSCs can affect the immune system. The higher percentage of PD-L1^+^ CSCs in patients with PD may confirm their resistance to conventional therapy, suggesting that CSCs may be an interesting target for immunotherapy.

**Electronic supplementary material:**

The online version of this article (10.1007/s00262-020-02648-y) contains supplementary material, which is available to authorized users.

## Introduction

The last years brought important progress in recognition of the role of the immune system in malignancy and the possibility of its modification thanks to new therapeutic strategies. The influence of the tumor microenvironment (TME) on the immune reaction in cancer is crucial. Lung cancer is one of the most aggressive solid tumors and carries a generally poor prognosis. Thus, the biology of this cancer is of interest. Lung cancer TME is composed of a large number of phenotypically and functionally different types of non-cancer cells including inflammatory cells, immune cells, vascular endothelial cells, fibroblast, smooth muscle cells, mesenchymal cells, adipocytes, and cancer cells along with cancer stem cells (CSCs) which represent a minor but significant population [1]. CSCs share some features with normal stem cells. In that, CSCs are capable of self-renewal, and they differentiate and give rise to the tumor cell heterogeneity that characterizes the complex architecture of solid tumors. These unique attributes underscore how CSCs can contribute to tumor genesis, aggressiveness, metastasis, and tumor recurrence following therapy [2]. Tumor cells have been shown to hamper and alter the immune system within the TME with different strategies to circumvent tumor cell recognition and killing [3]. Some data suggest that also CSCs may initiate mechanisms to circumvent a possible attack from the immune cells: loss of cancer antigen expression, activation of oncolytic pathways, and promotion of immunosuppressive milieu [4]. Unfortunately, this cross-talk between CSCs and other cells within the TME as well as interactions of CSCs with the immune system is not fully understood.

Lymph nodes (LNs) are common sites of metastasis and nodal disease predicts mortality in lung cancers. After the primary tumor site, metastatic LNs are the first place where tumor cells can induce immunosuppression [5]. Cancer cells that have metastasized to LNs must escape immune detection to avoid destruction. CD4^+^ T helper cells and the CD8^+^ cytotoxic T cells are the main effector cells that target cancer cells by controlling the humoral and cell-mediated response through the production of cytokines, perforin, and granzymes [6]. Priming and activation of tumor antigen-specific CD4^+^ and CD8^+^ lymphocytes occur in LNs; accordingly, LNs samples may be useful in studying cancer immunology [7, 8]. Immunosurveillance is the immune process of identifying and eliminating cancer cells [3]. Cancer cells evade an otherwise effective immune response through the expression of inhibitory molecules downregulating cytotoxic T-cell function [3]. In recent years, immunotherapy has been developed to strengthen cancer immunosurveillance. These monoclonal antibodies targeting checkpoint molecules PD-1 and PD-L1 have been especially effective in lung cancer. However, tumor PD-L1 immunohistochemistry (IHC) does not accurately predict response to these therapies [9]. Other receptors may serve as targets for agonist antibodies such as OX40, CD27, and CD28; conversely, additional antagonists against Tim3 or LAG-3 may help to promote tumor destruction [10]. Similar to primary TME, tumor cells in metastatic LNs shape their interactions with the host immune system by controlling the infiltration and reactivity of immune cells [6]. At present, little is known about the frequency of immunomodulatory molecules on CSCs as well as T cells in metastatic LNs. The immune cell composition in metastatic LNs may have predictive values for immune-based intervention. In our previous study, the presence of PD-L1^+^ CSCs in metastatic LNs in lung cancer patients was confirmed, which may suggest their importance in the cross-talk with PD-1^+^ immune cells and immunosuppressive properties [11].

The benefit of immune checkpoint inhibitors is achieved in about 50% of patients [12]; the predictive markers are widely investigated. The present study aimed to analyze the PD-L1^+^ CSCs in the context of T-cell subtypes frequencies and expression of immunomodulatory molecules in metastatic and non-metastatic LNs of non-small cell lung cancer (NSCLC) patients.

## Materials and methods

### Patients and specimen collection

We investigated consecutive enrolled treatment-naïve patients during a lung cancer diagnosis. The group consisted of 20 lung cancer patients. Patients form a new group, which had not been investigated in our previous studies. Patient characteristics are summarized in Supplementary Table 1. A chest computed tomography scan with intravenous contrast administration was performed before endobronchial ultrasound-guided biopsy (endobronchial ultrasound: EBUS/TBNA). The LNs’ involvement was classified according to the TNM 8th edition [13]. During EBUS, all LNs that were accessible for biopsy were punctured, beginning from the most distal TNM station. Only patients with histologically confirmed primary lung cancers were included in the study group. To ensure the quality of the material obtained during EBUS/TBNA procedure, the quality of all samples was assessed by an experienced pathologist. When the quality was appropriate, the material was divided for cytopathology staining, molecular testing, and flow cytometry analysis. LNs were considered metastatic when the standard cytopathology samples were classified as positive and provided a clear diagnosis of cancer. The sampled LNs were used only at the point “0”, during diagnostics. No re-staging was performed for the purpose of this study.

During the diagnostic procedure, the cancer samples of all patients were tested for the presence of the mutations using next-generation sequencing (NGS) technique (Ion GeneStudio S5 Prime System). A targeted NGS custom-made diagnostic panel AmpliSeq Library Kit 2.0–384 LV (Thermos Fisher Scientific, USA) was applied. The list of examined mutations is placed in Supplementary Table 2.

Three months after EBUS/TBNA procedure, a follow-up CT scan was performed. The RECIST (Response Evaluation Criteria In Solid Tumors) version 1.1 evaluation guidelines were applied to evaluate the best overall response. Treatment received by each patient is described in Supplementary table 1. According to types of chemo/RT offered to patients, all patients received a standard chemo-radiotherapy depending on the histological type of NSCLC.

### Flow cytometry analysis

LN aspirates were obtained during routine EBUS/TBNA procedure of lung cancer diagnosis. After diagnostic aspiration, the additional sample was taken for flow cytometry analysis. About 1 ml of LNs aspirate was diluted in 0.9% NaCl, collected in tubes containing K2EDTA, and processed for flow cytometry. Briefly, 200 μl LN aspirate and 2 μl specific monoclonal antibodies were added to each cytometry tube. After 15 min of incubation in the dark, at room temperature, erythrocytes were lysed with lysing solution for 10 min and washed with FACS flow solution. The cells were subsequently fixed in 200 μL of FACS Flow solution. Data were acquired using an LSR II flow cytometer (BD Biosciences) with FACS DivaTM software and analyzed with FlowJo version 9 (Tree Star Inc software, Ashland, OR, USA). Depending on yield, between 100,000 and 1,000,000 cells were used for CSCs and T-cell phenotyping by flow cytometry.

To identify CSCs in LNs aspirates, antibodies described in Supplementary Table 3 were applied. We defined CSCs cells as: CD45^−^/CD184^+^/EpCAM^+^/CD133^+^/CD44^+^/CD90^+^ [14–17]. Mature tumor cells were defined as: CD45^−^/CD184^+^/EpCAM^+^ [9, 18]. Additionally, antibody against PD-L1 was applied. To describe the lymphocyte phenotype in LNs’ aspirates, antibodies described in Supplementary table 4 were applied. Tregs were defined as CD4^+^/CD25^high^/CD127^low^.

### Statistical analysis

Mann–Whitney *U* test was performed to compare the differences between lymphocyte subpopulation in metastatic vs. non-metastatic LNs and the differences between PD-L1^+^ CSCs in patients with and without oncogene addiction; a *p* value of < 0.05 was considered statistically significant. Kruskal–Wallis test was performed to compare the differences between PD-L1^+^ CSCs in patients with progressive disease (PD), stable disease (SD), and partial response (PR). A *p* value of < 0.05 was considered statistically significant. Correlation analyses were performed by calculating the Pearson r coefficient. Differences were considered statistically significant when *p* < 0.05. All analyses were performed using Prism (Version 5, GraphPad Software, La Jolla, CA, USA).

## Results

### CSCs frequencies

Analysis of LNs’ aspirates revealed the presence of cancer cells in 18 samples (90%). The gating strategy of the cancer cells and CSCs is described in Supplementary Figure 1. CSCs and PD-L1^+^ CSCs were found in 17 samples (85%). Samples with more than 0.01% CSCs were regarded as positive (according to [14, 17]). Cancer cells, CSCs, and PD-L1^+^ CSCs were much higher in frequency in metastatic than in non-metastatic LNs. The highest percentage of PD-L1^+^ CSCs was observed in patients with a confirmed mutation—8.70%. There were no significant differences between the percentage of PD-L1^+^ CSCs in patients with and without confirmed mutations (data not shown). Patients with mutations were defined when any molecular aberration was confirmed. The patient number was too low for separate analysis for each mutation. Additionally, there were no significant differences between the CSCs and PD-L1^+^ CSCs and TNM stage. There were no significant differences between the frequency of PD-L1^+^ CSCs and TNM stage (data not shown). There were no significant differences between the frequency of PD-L1^+^ CSCs in patients with positive metastatic disease and without metastatic disease (Supplementary Figure 1.F).

### Frequencies of lymphocyte subsets and immunomodulatory molecules on lymphocyte subsets

**Fig. 1:**
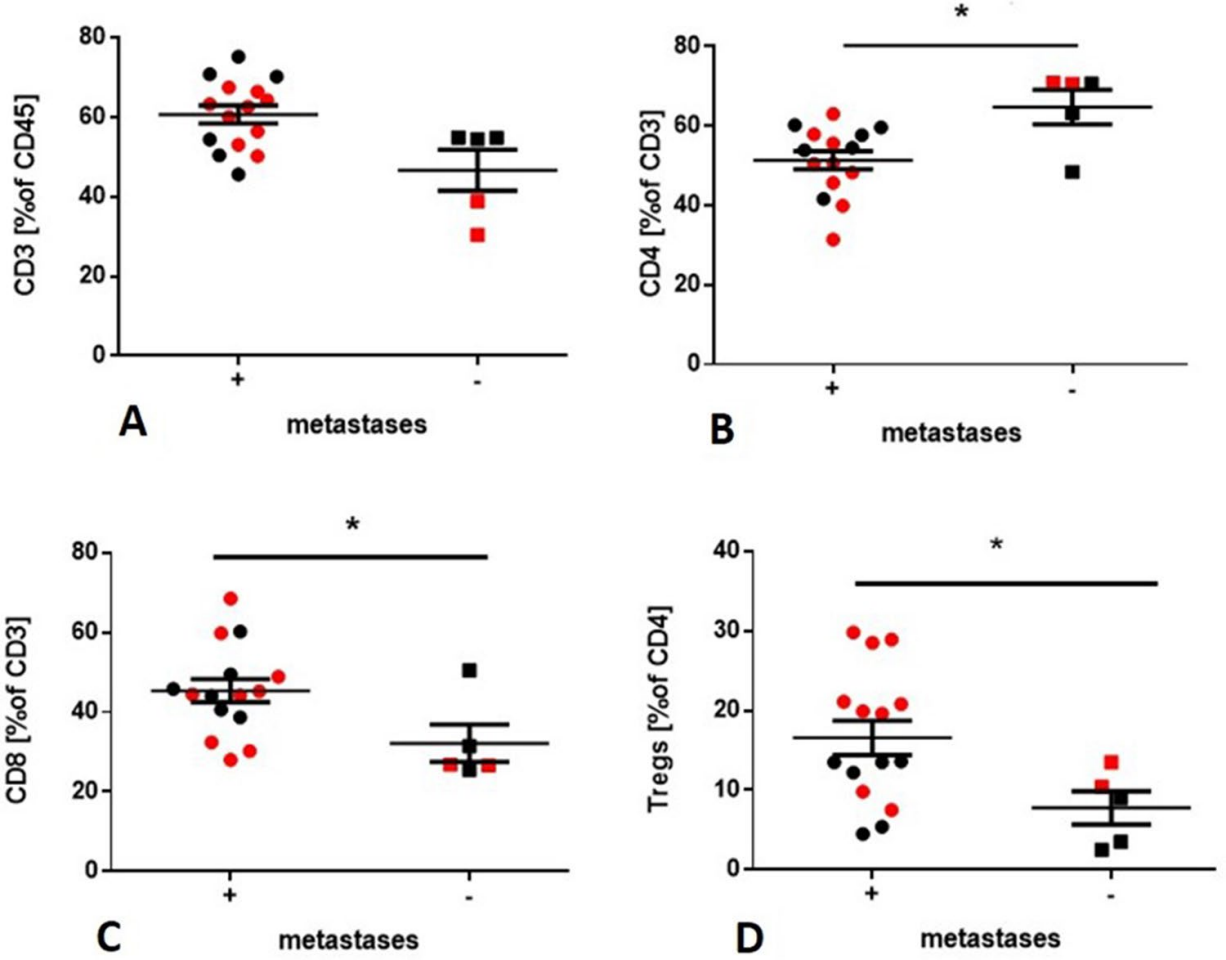
Differences in lymphocyte frequency in metastatic vs. non-metastatic LNs. Red dots are representative for patients with confirmed mutations. **a** Percentage of T cells described as a percentage of CD45^+^cells in metastatic vs. non-metastatic LNs. **b** Percentage of CD4^+^ T cells described as a percentage of CD3^+^ cells in metastatic vs. non-metastatic LNs *p = 0.014; 95% CI for difference: (46.53%, 56.19%) vs. (52.70%, 76.82%) **c** Percentage of CD8^+^ T cells described as a percentage of CD3^+^ cells in metastatic vs. non-metastatic LNs. *p = 0.418; 95% CI for difference: (39.16%, 51.59%) vs. (19.15%, 45.21%) **d** Percentage of Tregs described as a percentage of CD4^+^ T cells in metastatic vs. non-metastatic LNs. *p = 0.326; 95% CI for difference: (11,96%, 21.19%) vs. (1.98%, 13.58%)

**Fig. 2:**
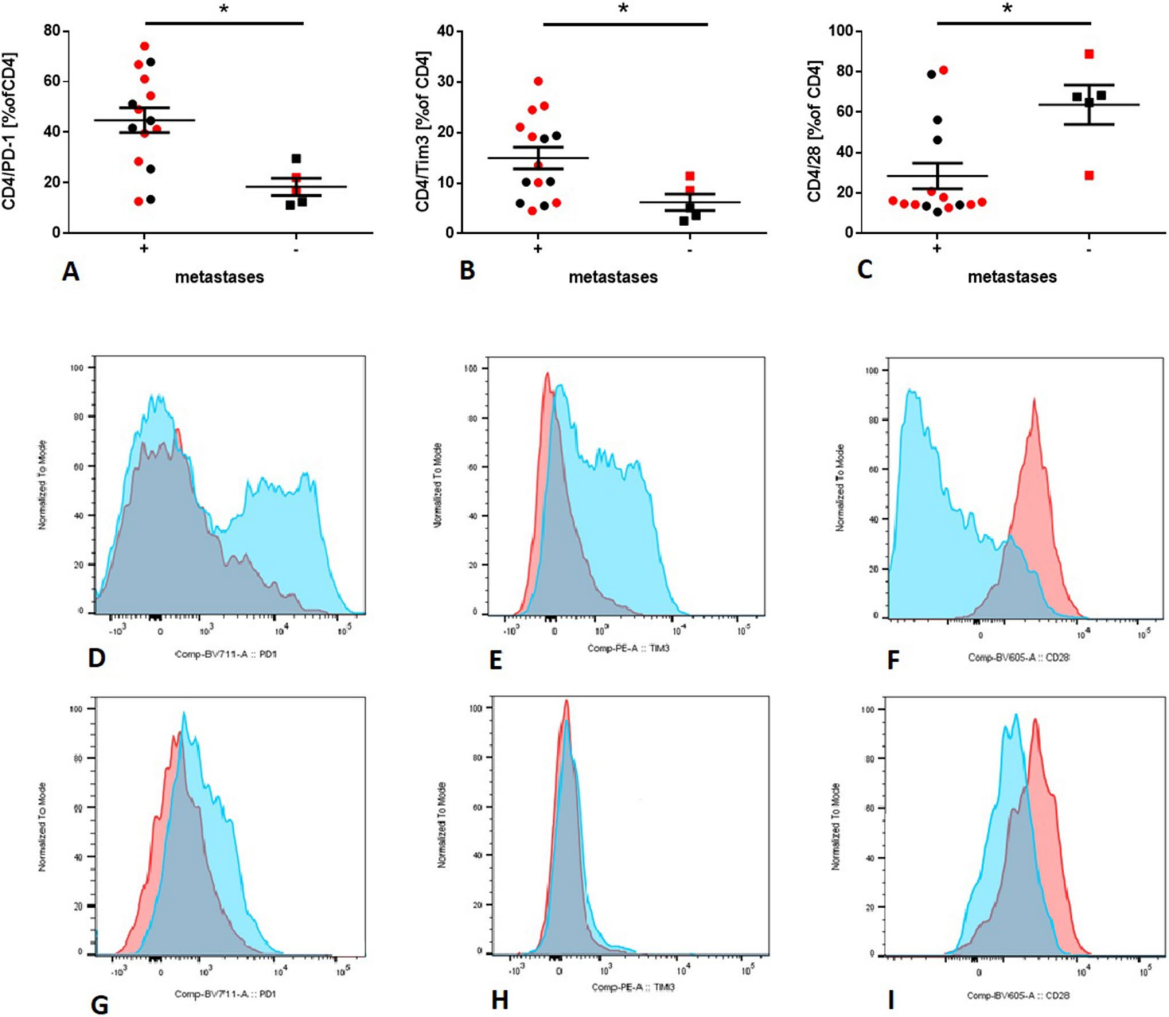
Frequencies of immunomodulatory molecules on lymphocyte subsets. Red dots are representative for patients with confirmed mutations. Figure. 4d–I shows representative LNs of the metastatic and non-metastatic patients. **a** The frequency of PD-L1^+^CD4^+^ T cells was significantly higher in metastatic LNs than in non-metastatic LNs. *p = 0.0036; 95% CI for difference: (37,41%, 57,49%) vs. (10,55%, 23,33%) **b** The frequency of Tim3^+^CD4^+^ T cells was significantly higher in metastatic LNs than in non-metastatic LNs. *p = 0.0193; 95% CI for difference: (8,44%, 18,18%) vs. (1,34%, 6,21%) **c** The frequency of CD28^+^CD4^+^ T cells was significantly lower in metastatic LNs than in non-metastatic LNs. *p = 0.0099; 95% CI for difference: (12.99%, 39.15%) vs. (37.27%, 92.09%) **d** Expression of PD1 on CD4^+^ T cells in metastatic LN (blue) and non-metastatic LN (red). **e** Expression of Tim3 on CD4^+^ T cells in metastatic LN (blue) and non-metastatic LN (red). **f** Expression of CD28 on CD4^+^ T cells in metastatic LN (blue) and non-metastatic LN (red). **g** Expression of PD1 on CD8^+^ T cells in metastatic LN (blue) and non-metastatic LN (red). **h** Expression of Tim3 on CD8^+^ T cells in metastatic LN (blue) and non-metastatic LN (red). **i** Expression of PD1 on CD8^+^ T cells in metastatic LN (blue) and non-metastatic LN (red)

Flow cytometry allowed distinguishing T-cell subpopulations: CD4^+^ T cells, CD8^+^ T cells, and Tregs (CD127^low^CD25^high^). Expression of OX40, CD27, CD28, Fas, PD-1, Tim3, and LAG3 on CD4^+^ T cells and CD8^+^ T cells was analyzed. The gating strategy is presented in Supplementary Figure 2. The frequency of total CD3^+^ T cells in metastatic and non-metastatic LNs was similar. However, metastatic LNs contained a lower percentage of CD4^+^ T cells than non-metastatic (51,36% vs. 64,76%, respectively, *p* = 0.0140), whereas the percentage of CD8^+^ T cells and Tregs were increased as compared to non-metastatic LNs (44,5% vs. 26.8%, *p* = 0.4180; 16.57% vs. 7.78%, *p* = 0.3216, respectively) (Fig. 1). CD4^+^ T cells in metastatic LNs had a higher frequency of PD1 (*p* = 0.0036) and Tim3 (*p* = 0.0193), but had a lower expression of CD28 than in non-metastatic LNs, (*p* = 0.0099) (Fig. 2). Non-significant differences were observed between the expression of immunomodulatory molecules on CD8^+^ T cells in metastatic vs. non-metastatic LNs. There were no significant differences between the T-cell phenotype and TNM stage. There were no significant differences between the T-cell phenotype in patients with positive metastatic disease and without metastatic disease.

### Correlation of the percentage of cancer cells, CSCs, and pd-l1^+^ CSCs with lymphocyte subsets and immunomodulatory molecules on lymphocyte subsets in metastatic LNs

**Fig. 3:**
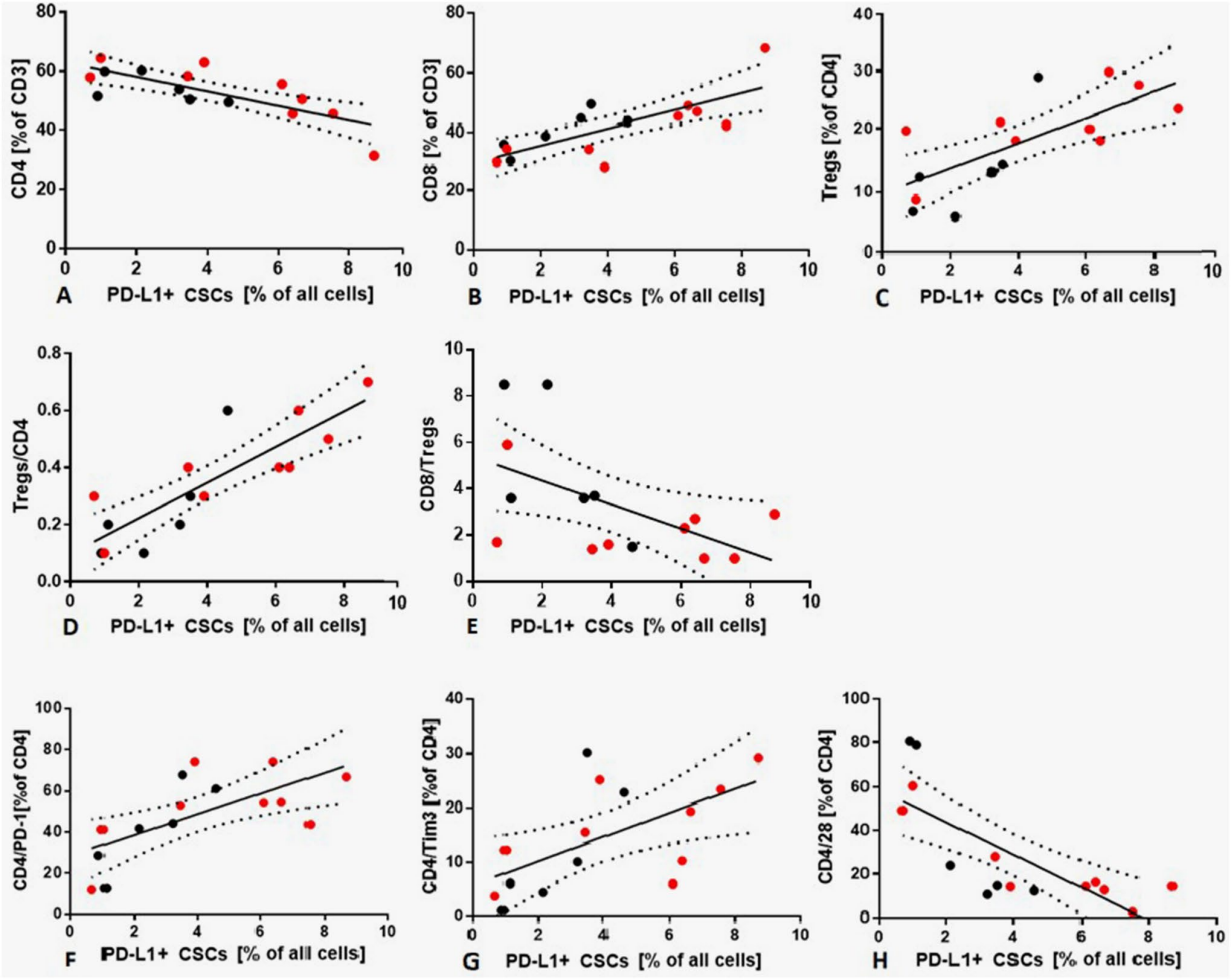
The correlations between PD-L1^+^ CSCs and T-cell subsets. Red dots are representative for patients with confirmed mutations. **a** PD-L1^+^ CSCs are negatively correlated with CD4^+^ T cells (*r* = − 0.7539, *p* = 0.0009). **b** PD-L1^+^ CSCs are positively correlated with CD8^+^ T cells (*r* = 0.7583, *p* = 0.0011). **c** PD-L1^+^ CSCs are positively correlated with Tregs (*r* = 0.7239, *p* = 0.0023). **d** PD-L1^+^ CSCs are positively correlated with Tregs/CD4 ratio. **e** PD-L1^+^ CSCs are negatively correlated with CD8/Tregs ratio. **f** PD-L1^+^ CSCs are positively correlated with PD-1^+^ CD4^+^ T cells (*r* = 0.7246, *p* = 0.0042). **g** PD-L1^+^ CSCs are positively correlated with Tim-3^+^ CD4^+^ T cells (*r* = 0.7126, *p* = 0.0058). **h** PD-L1^+^ CSCs are negatively correlated with CD28^+^ CD4^+^ T cells (*r* = − 0.7539, *p* = 0.0009)

The percentage of cancer cells was positively correlated with the percentage of CD8^+^ T cells (*r* = 0.6025, *p* = 0.0174), Tregs (*r* = 0.5317, *p* = 0.0436), and negatively correlated with the percentage of CD4^+^ T cells (*r* = − 0.5989, *p* = 0.0303 (Supplementary Figure 3). The percentage of CSCs was negatively correlated with the percentage of CD4^+^ T cells (*r* = − 0.6320 *p* = 0.0253) (Supplementary Figure 3). The percentage of PD-L1^+^ CSCs was positively correlated with the percentage of CD8^+^ T cells (*r* = 0.7583, *p* = 0.0011) (Fig. 3b), Tregs (*r* = 0.7239, *p* = 0.0023) (Fig. 3c), PD-1^+^ CD4^+^ T cells (*r* = 0.7246, *p* = 0.0042) (Fig. 3f), and Tim3^+^ CD4^+^ T cells (*r* = 0.7126, *p* = 0.0058) (Fig. 3g), whereas PD-L1^+^ CSCs negatively correlated with CD4^+^ T cells (Fig. 3a) and CD28^+^ CD4^+^ T cells (*r* =− 0.7539, *p* = 0.0009 and *r* = − 0,7963, *p* = 0.0004, respectively) (Fig. 3h). PD-L1-negative CSCs correlated only negatively with CD4^+^ T cells (*r* = − 0.6842, *p* = 0.0191) (data not shown). To strengthen our analysis, we correlated the proportion of cancer cells, PD-L1^+^ cancer cells, PD-L1^−^ CSCs, and PD-L1^+^ CSCs in all cancer cells with the proportion of different lymphoid populations (Supplementary Figure 4). The percentage of cancer cells positively correlated with the percentage of Tregs (*r* = 0.5862, *p* = 0.0362). PD-L1^+^ cancer cells positively correlated with Tregs (*r* = 0.5885, *p* = 0.0291). Analysis did not reveal any significant correlation between PD-L1^−^ CSCs and lymphocyte subsets. Considering PD-L1^+^ CSCs, we found that the PD-L1^+^ CSCs positively correlated with the percentage of CD8^+^ T cells (*r* = 0.6225, *p* = 0.0298), Tregs (*r* = 0.6257, *p* = 0.0280), PD-1^+^ CD4^+^ T cells (*r* = 0.6474, *p* = 0.0233), and Tim3^+^ CD4^+^ T cells (*r* = 0.6161, *p* = 0.0198). PD-L1^+^ CSCs negatively correlated with CD4^+^ T cells and CD28^+^ CD4^+^ T cells (*r* = − 0.7243, *p* = 0.0095, and *r* = − 0.6204, *p* = 0.0236 respectively).

### Percentage of PD-L1^+^ CSCs and CD4 T-cell phenotype in LNS and response to treatment

**Fig. 4:**
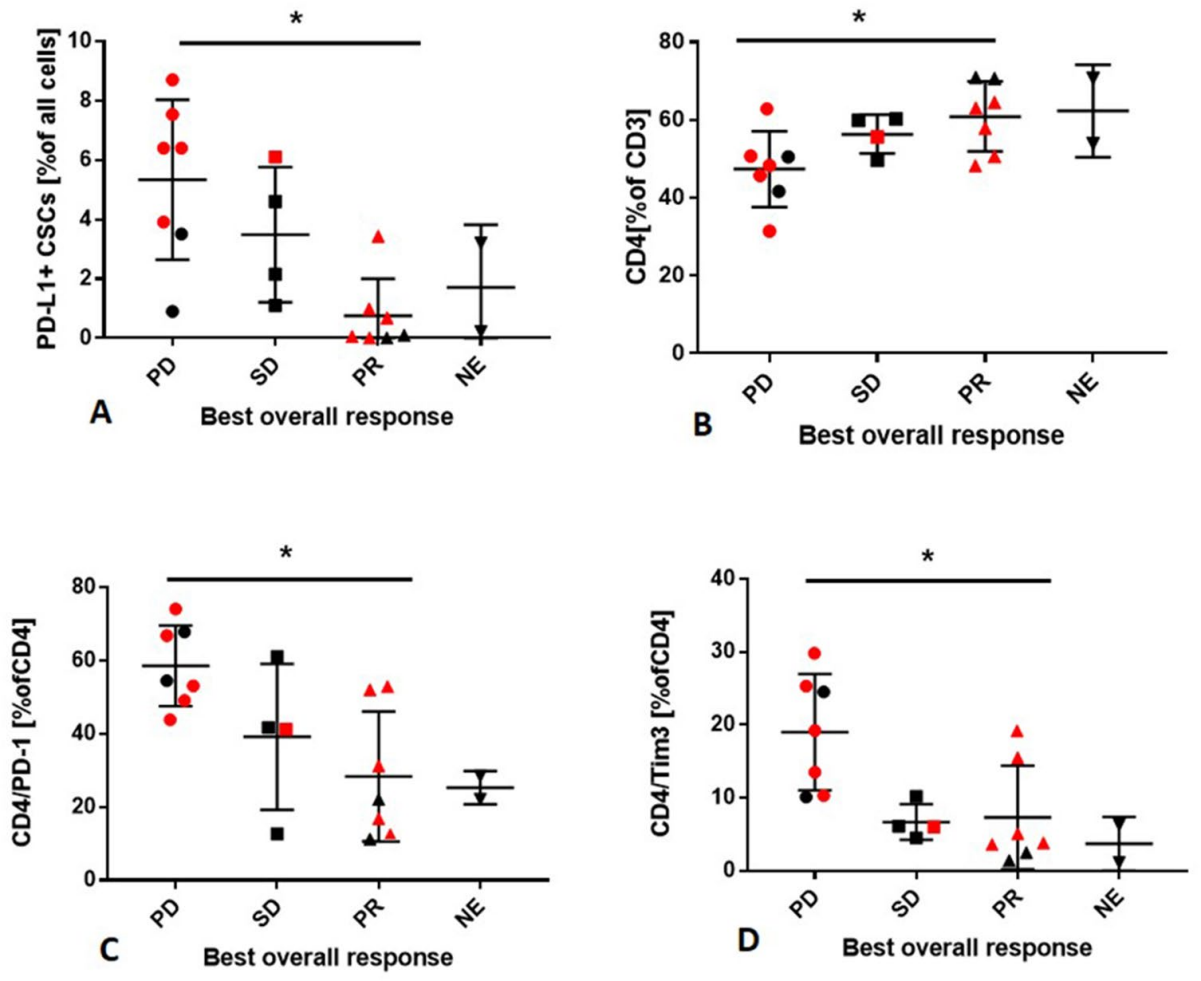
Distribution of PD-L1^+^ CSCs and CD4^+^ T cells in NSCLC patients depending on the best overall response. **a** Frequency of PD-L1^+^ CSCs differs in patients with PD vs. SD vs. PR. *p = 0.0014 95% CI for difference: (2.85%, 7.82%) vs. (1.13%, 7.11%) vs. (0.39%, 1.90%). **b** Frequency of CD4 T cells in patients with PD vs. SD vs. PR. *p = 0.0453 95% CI for difference: (38.38%, 56.31%) vs. (48.45%, 64.2%) vs. (52.55%, 69.14%). **c** Frequency of PD-1^+^ CD4^+^ T cells in patients with PD vs. SD vs. PR. *p = 0.0099 95% CI for difference: (48.40%, 68.74%) vs. (7.45%, 70.89%) vs. (11.98%, 44.79%). **d** Frequency of Tim-3^+^ CD4^+^ T cells in patients with PD vs. SD vs. PR. *p = 0.0125 95% CI for difference: (11.65%, 26.38%) vs. (2.81%, 10.59%) vs. (0.77%, 13.89%)

Next, we investigated whether the cancer cells, CSCs, or T-cell phenotype was associated with clinical response in NSCLC patients. Of all 20 patients, seven patients (35%) had progressive disease, four patients (20%) were described as stable disease (SD), and seven patients (35%) as partial response (PR). In two patients, the follow-up was missing, so they were qualified as not evaluable. The statistical analysis revealed significant differences in the percentage of PD-L1^+^ CSCs between patients with PD vs. SD vs. PR (*p* = 0.0014) (Fig. 4a). There were no significant differences in the percentage of cancer cells, PD-L1^+^ cancer cells, or PD-L1^−^ CSCs between PD, SD, and PR patients. Among T cells, only a percentage of CD4^+^ T cells was associated with prognosis. PD-1^+^ CD4^+^ T cells and Tim3^+^ CD4^+^ T cells were higher in patients with PD as compared to patients with SD or PR (Fig. 4.b–d). There were no differences in Tregs and CD8^+^ T-cell frequency and CD8^+^ T-cell phenotype between patients with PD, SD, or PR.

## Discussion

The interaction between CSCs and the immune system is not well understood and is currently of much interest. Previously, the expression of PD-L1 on putative CSCs (EpCAM^+^/CD133^+^) was confirmed in LN aspirates in NSCLC patients by flow cytometry [11]. These properties of PD-L1^+^ CSCs suggest their immunogenic potential and encouraged us to investigate whether there is an association with these particular cells and the phenotype of lymphocyte. In the present study, we confirmed the utility of routine EBUS/TBNA procedure to evaluate immune cell composition. Using multiple markers, we confirmed the identification of CSCs and we demonstrated that PD-L1^+^ CSCs strongly associate with an altered T-cell phenotype and especially the frequency of regulatory molecules expressing T cells in metastatic LNs of NSCLC patients. It might indicate that similarly to primary tumors, CSCs shape their interactions with the host immune system by controlling the reactivity of immune cells. Results of the current study confirmed the utility of EpCAM, CD184, CD44, CD133, and CD90 as markers of lung CSCs. So far, many markers, signatures, and methods have been used to evaluate the phenotype of lung CSCs [4, 19]. To the best of our knowledge, our study is the first one, to evaluate different putative CSCs markers simultaneously using flow cytometry.

First, we found that the percentage of cancer cells correlates with the percentage of CD8^+^ T cells, CD4^+^ T cells, and Tregs. It has been proven that metastatic LNs often fail to produce effective antitumor immunity and instead tolerize the patient to tumor antigens [20]. Cancer cells establish local and systemic immunosuppression via a variety of mechanisms. There have been immense advances in the understanding of the dynamic process of immunoediting in the TME and its prognostic significance. The most studied mechanism in NSCLC is the expression of PD-L1 that drives CD8^+^ T-cell exhaustion. However, less is known about changes at the level of the LNs. Research by other authors showed the feasibility of EBUS/TBNA samples of LNs for flow cytometric analysis of mature tumor cells and lymphocytes [8]. Similar to our findings, they reported a decrease in CD4^+^ T cells and an increase in CD8^+^ T cells in metastatic LNs compared to non-metastatic LNs. Therefore, it seems that the presence of a high number of tumor cells in the LN may diminish CD4^+^ T cells. However, little is known about the interaction between CSCs and Tregs specifically. In our study, the percentage of PD-L1^+^ CSCs correlates with the percentage of CD8^+^ T cells, CD4^+^ T cells, and Tregs, but not to the total frequency of T cells. Altogether, it seems that the frequency of CD8^+^ T cells, CD4^+^ T cells, and Tregs may be affected by the presence of cancer cells. However, the correlations between PD-L1^+^ cancer cells and T cells are stronger than cancer cells and T cells what may suggest the immunosuppressive potential of PD-L1^+^ CSCs.

In our group, 8/15 (53%) patients with confirmed LNs metastasis were classified as adenocarcinoma (ADC) and 2/15 (13%) were classified as adenosquamous. The highest percentage of PD-L1^+^ CSCs were observed in ADC patients. Unfortunately, this cohort of patients is insufficiently powered to detect whether significant changes in PD-L1^+^ CSCs’ frequencies could be observed between these histological subtypes. It has been shown that higher percentages of CD8^+^ T cells in metastatic LNs and lower CD8/Treg ratio are associated with ADC subtype [7, 21], what is in concordance with our results. Altogether, it seems that ADC may have a unique immune signature.

We found significant differences between immunomodulatory molecules: Tim3, PD-1, and CD28 on CD4 T cells between metastatic and non-metastatic LNs. Tim3^+^ CD4^+^ T cells and PD-1^+^ CD4^+^ T cells were correlated with increased frequencies of PD-L1^+^ CSCs, whereas CD28^+^ CD4 T cells were correlated with decreased frequencies of PD-L1^+^ CSCs. Interestingly, in our study, the highest CD28 percentage was observed in patients without confirmed metastases, but with low CSCs frequency (0,01–0,25%), which could be an indication of micrometastases. We suppose that high CD28 expression may be due to CD4^+^ T-cell activation related to micrometastases in LNs in lung cancer patients and may control the metastatic cells in these compartments. A low frequency of stemness markers was described in LNs in gastric cancer patients and was an independent predictive factor for LNs metastasis [22].

Correlation between PD-L1^+^ CSCs and PD1^+^ CD4^+^ T cells as well as Tim3^+^ CD4^+^ T cells suggest that CSCs interact with T cells, in an especially inhibitory setting and promote CD4^+^ T-cell anergy. Interestingly, these dependencies were not observed in the context of the cancer cells. It may suggest that the PD-L1^+^ CSCs may be specifically responsible for CD4^+^ T cells anergy. T‐cell exhaustion has been intensively discussed regarding CD8^+^ T cells, whereas the role of exhausted CD4^+^ T cells in the TME has not been fully evaluated. With chronic tumor antigen exposure, T cells in LNs can become progressively exhausted. Newly suppressed T cells express low amounts of PD-1 and are recoverable on treatment with anti-PD-L1/PD-1 therapies, whereas hyperexhausted T cells expressing high levels of PD-1 as well as other activation markers such as LAG-3 or Tim3 may be unrecoverable [20]. A similar analysis of the lymphocyte phenotype in EBUS/TBNA samples was performed by Van de Ven et al. [23]. Consistent with our study, they reported a higher frequency of Tregs and PD1^+^ CD4^+^ T cells in metastatic LNs. Tim3^+^ CD4^+^ T cells co-expressing PD-1 and exhibiting defects in proliferation and effector cytokine production were found in primary NSCLC TME [24]. These authors demonstrate that Tim3 expression on CD4 T cells but not on CD8^+^ T cells correlated with the presence of nodal metastases and advanced lung cancer stage [24]. In all, it seems that during NSCLC development not only the immunological composition of primary TME but also metastatic sites, such as metastatic LNs, are affected. Altogether, it seems that not only PD-1 but also other immunomodulatory molecules, such as Tim3, may serve as receptor targets, which requires more extensive research. A better understanding of the specialized functions of these receptors will inform the rational application of therapies that target these receptors.

Interestingly, another correlation study in NSCLC demonstrated a strong association between EMT (epithelial–mesenchymal transition) and an inflammatory TME with the expression of immune checkpoint molecules (PD-1, Tim3, LAG-3, PD-L1) [25]. Collectively, these findings suggest that EMT profoundly alters the susceptibility of cancer cells to immune surveillance. Among the CSCs’ regulation pathways, EMT is of particular interest as it enriches CSCs, is a key step toward metastasis, and has been proposed as a major mechanism of CSCs resistance including immunosurveillance [26]. The EMT process is described as independent of the mutational burden [25]. In our study, we have found that the highest percentage of PD-L1^+^ CSCs was observed in patients with oncogene addiction, but the percentage of PD-L1^+^ CSCs do not significantly differ between patients with and without confirmed mutations. Unfortunately, in our study, we do not investigate the expression of EMT associated regulators in EBUS/TBNA samples. An insufficient number of pre-clinical and clinical studies on CSCs in NSCLC patients make it impossible to evaluate whether the presence of CSCs is a result of EMT or high mutation burden, but it can create the direction of further studies.

Finally, we found that the percentage of PD-L1^+^ CSCs and CD4^+^ T-cell phenotype differs between patients with different responses to treatment. Although these results may be influenced by the small number of patients in each group, it may indicate that both PD-L1^+^ CSCs and CD4^+^ T cells could be indicative of rapid progression and poor prognosis in NSCLC patients. CSCs are largely resistant to treatments, which allows them to elude standard chemo- and radiotherapies. In our study, 9/15 metastatic patients with PD and SD were treated with chemo or combined chemo-radiotherapy. Of that nine patients, seven do not respond to this form of treatment. It may confirm the CSCs’ resistance to conventional treatments. Interestingly, we observed a patient with PR with metastatic disease treated with immunotherapy and TKI. Furthermore, it is possible that the treatment of the patients harboring PD-L1^+^ CSCs and PD-1^+^ CD4 T cells with anti-PD-1 or anti-PD-L1 therapies could improve their outcome. A similar finding was made by Chou et al. who reported that high levels of PD-L1 and CSCs correlate with the worst survival in pancreatic cancer patients [27]. Both in our and pancreatic cancer study, only the PD-L1^+^ population of CSCs was linked with more aggressive disease resistant to conventional treatment.

The major weakness of our study is the low sample size. We are aware that these findings should be confirmed or further investigated in larger patient groups. Nevertheless, our results confirm the utility of flow cytometric analysis of EBUS/TBNA samples to assess the interaction between CSCs and the immune system and their immunogenic potential in individual patients. Considering that PD-L1^+^ CSCs may contribute to NSCLC aggressiveness and resistance to conventional therapies, they may serve as a potential factor for prognostic evaluation of NSCLC and help in designing promising immune-based therapeutic strategies.

### Electronic supplementary material

Below is the link to the electronic supplementary material.

Supplementary file1 (PDF 691 kb)
