# Supplementary material for: PD-L1+ lung cancer stem cells modify the metastatic lymph-node immunomicroenvironment in nsclc patients
Source: Cancer Immunol Immunother. 2020 Aug 17;70(2):453–61. doi: 10.1007/s00262-020-02648-y (PMC7889682; doi:10.1007/s00262-020-02648-y)
Supplement: Supplementary file 1 — Supplementary file1 (PDF 691 kb) [file 262_2020_2648_MOESM1_ESM.pdf]

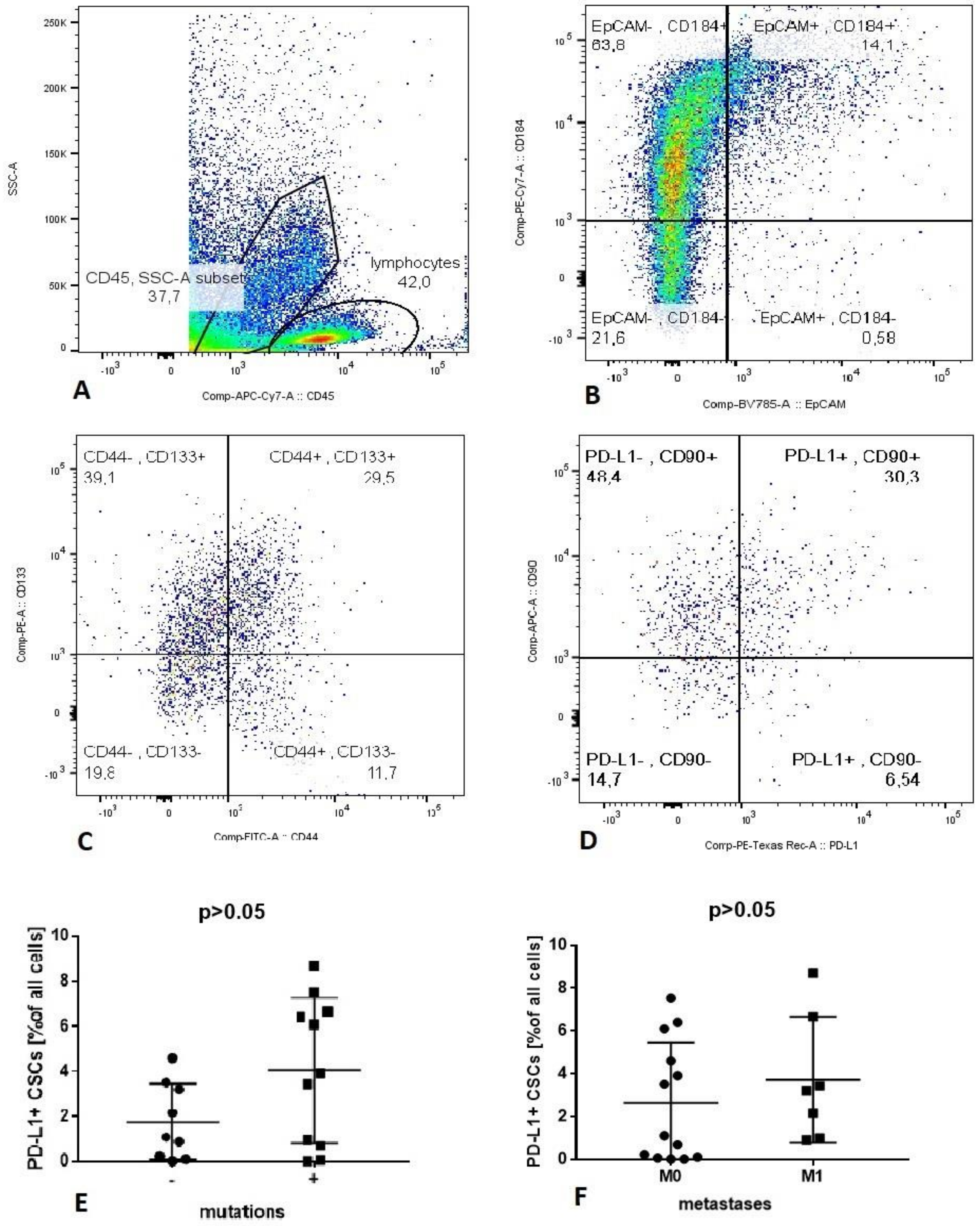

Supplementary Figure 1. The gating strategy of cancer cells and CSCs in LNs aspirates. A) The population of CD45- cells (non-lymphoid origin). B) CD45-/CD184+/EpCAM+ gate – cancer cells (14,1% of all cells). C) Among cancer cells 29,5% cells express markers of stemness: CD44 and CD133. D) Among CD45-/CD184+/EpCAM+/CD44+/CD133+ cells 75% of cells express additional marker of stemness CD90. CSCs represent 3,5% of all cells in LN aspirate. Finally, 36,5% of CSCs are PDL-1+. PDL-1+. CSCs represent 1,5% of all cells in LN aspirate. E) The highest frequencies of PD-L1+ CSCs were observed in patients with confirmed mutations (p=0.465; not significant) F) The frequencies of PD-L1+ CSCs in patients with positive metastatic disease versus patients with no metastatic disease.

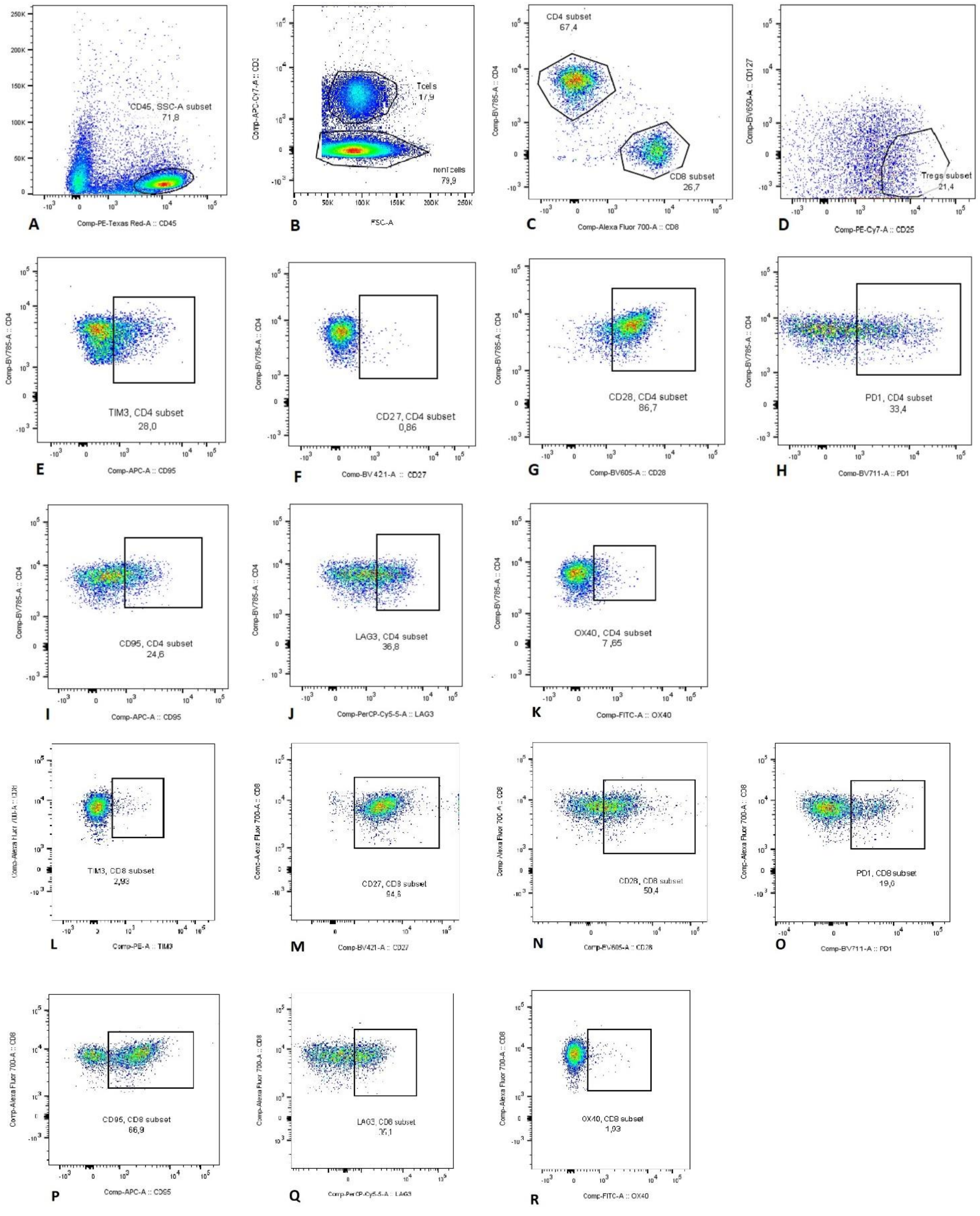

Supplementary Figure 2. Lymphocyte gating strategy. A-D) Distinguishing main lymphocyte subsets: T cells, CD4 T cells, CD8 T cells and Tregs. E-K) Gating CD4+ T cells positive for immunomodulatory molecules. L-R) Gaiting CD8+ T cells positive for immunomodulatory molecules.

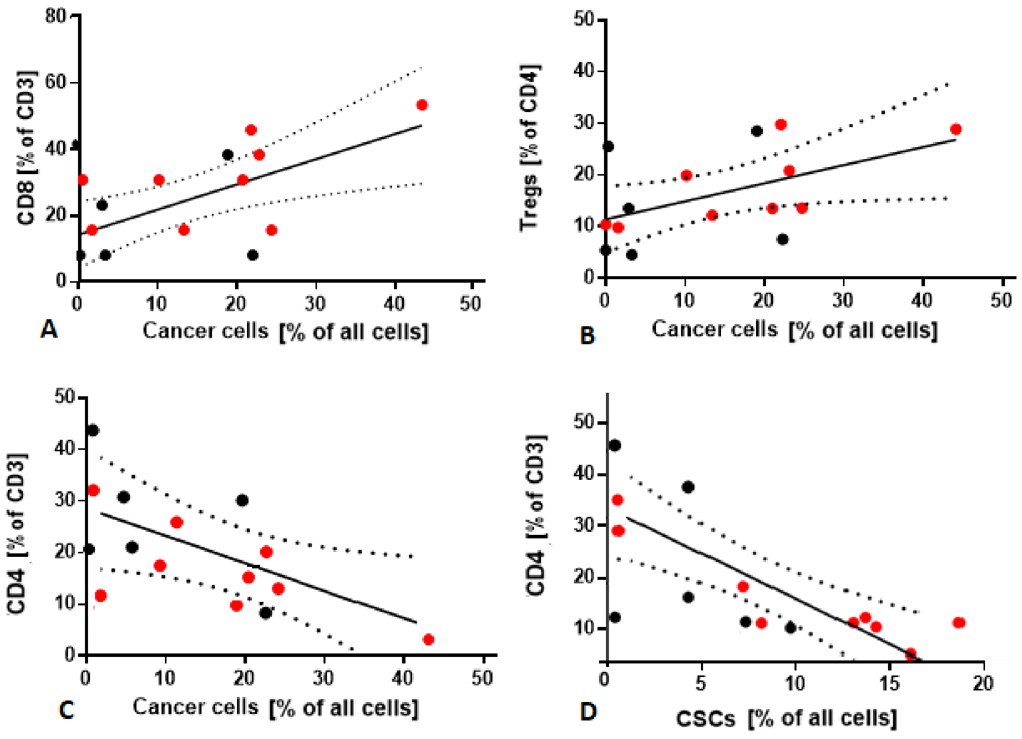

Supplementary figure 3. Correlations between cancer cells, CSCs and T cell subsets in metastatic LNs. Red dots are representative for patients with confirmed mutations. A) Cancer cells are positively correlated with CD8 T cells. r=0.6025, p=0.0174. B) Cancer cells are positively correlated with Tregs r=0.5317, p=0.0436. C) Cancer cells are negatively correlated with CD4 T cells. r=-0.5989, p=0.0303 D) CSCs are negatively correlated with CD4 T cells r= -0.6320 p=0.0253

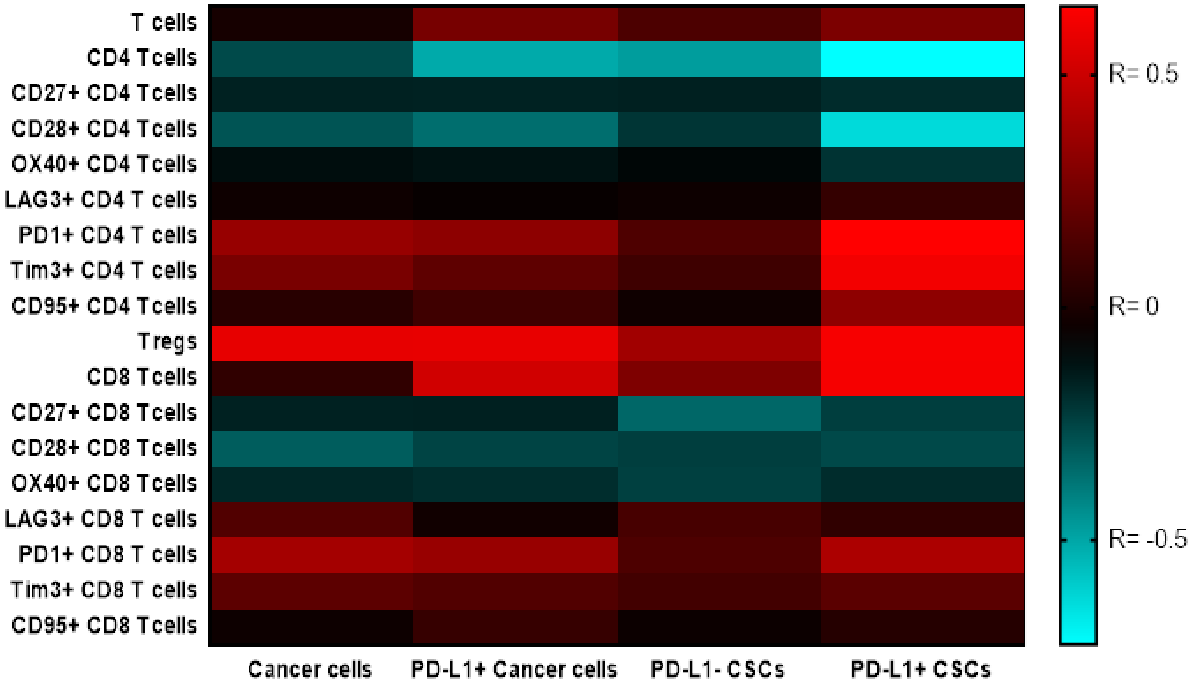

Supplementary figure 4 . A heatmap of Pearson correlation coefficients in R values over all investigated lymphocyte subpopulations and cancer cells subpopulations (described as a percentage of all cancer cells). The percentage of cancer cells positively correlated with the percentage of Tregs (r=0.5862, p= 0.0362). PD-L1+ cancer cells positively correlated with Tregs (r=0.5885, p=0.0291) and CD8 T cells (r=0.52677). PD-L1+ CSCs correlated positively with the percentage of CD8+ T cells (r=0.6225, p=0.0298), Tregs (r=0.6257, p=0.0280), PD-1+ CD4+ T cells (r= 0.6474, p=0.0233), and Tim3+ CD4+ T cells (r= 0.6161, p=0.0198). PD-L1+ CSCs negatively correlated with CD4+ T cells and CD28+ CD4+ T cells (r= -0,7243, p=0.0095 and r= -0,6204, p=0.0236 respectively).

	metastases	Histological subtype	TNM	Stage	Sex	Age	NGS result	Pack years	treatment	follow-up
1.	0	SQCLC	T4N0M0	IIIC	F	59	0	80 (ex)	C-R	NE
2.	0	NOS	T1bN1M0	IIB	F	64	0	0	C	PD
3.	0	ASC	T2N1M0	IIB	M	65	0	30	C	PR
4.	0	ADC	T2bN0M0	IIA	M	63	KRAS exon2: c.38G>A,	70	none	PR
5.	0	ADC	T1aN0M0	IA	M	56	PIK3CA c.3140A>G, p.H1047R	50	none	PR
6.	1	SQCLC	T2N2M1	IV	M	72	0	0	C	PD
7.	1	SQCLC	T4N3M0	IIIC	M	69	0	64 (ex)	C	PD
8.	1	ADC	T4N1M1	IV	M	78	TP53 exon 5: c.473G>T; p.R158L<br>HER2 amplification	70	C-R	PD
9.	1	ADC	T4N1M0	IIIA	F	65	KRAS exon 2: c.34G>T; p.G12C<br>TP53 exon 6: c.594del; p.G199Efs*48	90	C-I	PD
10.	1	ADC	T3N3M0	IIIB	F	40	HER2 exon 20: c.2313_2324dup; p.Y772_A775dup	0	C-I	PD
11.	1	ADC	T4N1M0	IIIA	F	76	KRAS exon 2 + EGFR exon 21 c.2573T>G	60	TKI	PD
12.	1	ADC	T4N2M1	IV	F	71	KRAS exon 2: c.35G>C;	60	C-R	PR
13.	1	ADC	T4N3M1	IV	M	71	MET exon 14: c.3082+1_3082+3delinsTT; p.?, MET exon 19: c.3736G>A; p.D1246N.	50 (ex)	I	PR
14.	1	ADC	T2aN2M1	IV	M	63	EGFR ex19; c.2235_2249del; p.Glu746_Ala750del	50	TKI	PR
15.	1	LCNEC	T1N2M0	IIIA	M	86	TP53 exon 8: c.845G>C; p.R282P<br>TERT promotor: n.*1095475G>A, GNAQ exon 5: c.674C>G; p.S225	0	C	PR
16.	1	SQCLC	T2aN2M1a	IVA	M	62	0	30	C	SD
17.	1	SQCLC	T2aN1M0	IIB	M	77	0	80	C	SD
18.	1	ADC	T2aN3M0	IIIB	F	58	0	0	C	SD
19.	1	ADC	T3N3M0	IIIC	F	70	HER2exon 20: A775_G776insYVMA	50	C	SD
20.	1	ASC	T3N2M1	IV	M	73	0	48	none	NE

Supplementary Table 1. Patient characteristics. PR = partial response, SD = stable disease, PD = progressive disease, NE = not evaluable; C = Chemotherapy, TKI = tyrosine kinase inhibitor, I = immunotherapy, C-I = combined chemo- and immunotherapy, C-R = combined chemo- and radiotherapy; ADC- adenocarcinoma, SQCLC- squamous cell carcinoma, ASC- adenosquamous carcinoma, LCNEC- large cell neuroendocrine carcinoma, NOS- not otherwise specified

Coding sequence	CDKN2A	
	PTEN	
	TP53	
Mutation hotspots	AKT1 (exon 3)	IDH2 (exon: 4)
	ALK (exon: 20, 22-25)	KIT (exon: 8, 9, 11, 13, 14, 17)
	APC (exon 14)	KRAS (exon: 2,3,4)
	ARAF (exon 7)	MAP2K1 (exon: 2 and 3)
	BRAF (exon: 11, 15)	MET (exon: 2, 14, 19)
	CTNNB1 (exon: 3, 7, 8)	MYD88 (exon 5)
	EGFR (exon: 18-21)	NOTCH1 (exon: 26, 27)
	HER2 (exon: 19-21)	NRAS (exon: 2,3,4)
	EZH2 (exon: 16)	PDGFRA (exon: 12, 14, 18)
	FBWX7 (exon: 9, 10)	PIK3CA (exon: 10 and 21)
	FGFR1 (exon: 4, 7, 12)	POLD1 (exon: 12)
	FGFR2 (exon: 7, 9, 12)	POLE (exon: 9, 13)
	FGFR3 (exon: 7, 9)	RAF1 (exon: 7)
	FOXL2 (exon 1)	RET (exon: 11, 16)
	GNA11 (exon: 4, 5)	RNF43 (exon: 3, 4, 9)
	GNAQ (exon: 4, 5)	ROS1 (exon: 38, 41)
	GNAS (exon: 8, 9)	SMAD4 (exon: 3, 9, 12)
	HRAS (exon: 2,3,4)	STK11 (exon: 4, 5, 8)
	IDH1 (exon: 4)	
In situ hybridization	NTRK1	
	ROS1	
	RET	
	MET	
Immunohistochemistry	ALK gene rearrangement	

Supplementary Table 2. Targeted NGS Custom made diagnostics V4 panel

Target	Conjugate	Company	Clone
CD44	FITC	BD Biosciences	L178
CD133	PE	BD Biosciences	W6B3
CD184 (CXCR4)	PE-Cy7	BD Biosciences	I2G5
CD90	APC	BD Biosciences	5E10
CD326 (EpCAM)	BV785	Biolegend	9C4
CD45	APCeF700	eBioscience	HI30
PD-L1	PeCf594	BD Biosciences	M1H1

Supplementary Table 3. Antibodies used for flow cytometry identification of PD-L1+ CSCs

Target	Conjugate	Company	Clone
CD45	PeCf594	BD Biosciences	HI30
CD3	APCeF700	eBioscience	UCHT1
CD8	AF700	Biolegend	SK1
CD4	BV785	BD Biosciences	M-A251
CD134 (OX40)	FITC	BD Biosciences	ACT35
CD366 (TIM3)	PE	Biolegend	F38-2E2
CD223 (LAG3)	PerCp-Cy5.5	Biolegend	C9B7W
CD95 (FAS)	APC	eBioscience	DX2
CD27	BV421	BD Biosciences	M-T272
CD28	BV605	BD Biosciences	CD28.2
CD127	BV650	BD Biosciences	hIL7R-M21
CD279 (PD-1)	BV711	BD Biosciences	EH12.1

Supplementary Table 4. Antibodies used for flow cytometry analysis of lymphocyte repertoire.
